# Priorities for health and wellbeing for older people with and without HIV in Uganda: a qualitative methods study

**DOI:** 10.1002/jia2.26000

**Published:** 2022-09-29

**Authors:** Zahra Reynolds, Rebecca Gilbert, Ruth Sentongo, Ana‐Claire Meyer, Deanna Saylor, Samson Okello, Noeline Nakasujja, Meredith Greene, Janet Seeley, Alexander C. Tsai, Stephen Asiimwe, Lien Quach, Brianne Olivieri‐Mui, Mark J. Siedner

**Affiliations:** ^1^ Medical Practice Evaluation Center Massachusetts General Hospital Boston Massachusetts USA; ^2^ Mbarara University of Science and Technology Mbarara Uganda; ^3^ Johns Hopkins University School of Medicine Baltimore Maryland USA; ^4^ University Teaching Hospital Lusaka Zambia; ^5^ Gillings School of Global Public Health University of North Carolina Chapel Hill North Carolina USA; ^6^ Makerere University Kampala Uganda; ^7^ University of California San Francisco California USA; ^8^ London School of Hygiene and Tropical Medicine London UK; ^9^ Harvard Medical School Boston Massachusetts USA; ^10^ Kabwohe Clinical Research Centre Kabwohe Uganda; ^11^ University of Massachusetts Boston Massachusetts USA; ^12^ Northeastern University Boston Massachusetts USA

**Keywords:** HIV, ageing, non‐communicable disease, qualitative, sub‐Saharan Africa, quality of life

## Abstract

**Introduction:**

With improved HIV treatment availability in sub‐Saharan Africa, the population of older people with HIV (PWH) is growing. In this qualitative study, we intended to understand (1) the lived experiences of ageing people in rural Uganda, with and without HIV, (2) their fears and health priorities as they grow older.

**Methods:**

We conducted 36 semi‐structured interviews with individuals with and without HIV in Mbarara, Uganda from October 2019 to February 2020. Interview guide topics included priorities in older age, physical functioning in daily activities, social functioning, HIV‐related stigma and the impact of multimorbidity on health and independence. Interviews were conducted in Runyankole, transcribed, translated and inductively coded thematically by two researchers with tests for inter‐coder reliability.

**Results:**

The respondents were purposively sampled to be evenly divided by sex and HIV serostatus. The median age of respondents was 57 (49–73). Two‐thirds were married or cohabitating, 94% had biological children and 75% cited farming as their primary livelihood. Overall, PWH considered themselves as healthy or healthier than people without HIV (PWOH). PWH rarely considered their HIV status a barrier to a healthy life, but some reported a constant sense of anxiety as it relates to their long‐term health. Irrespective of HIV status, nearly all respondents noted concerns about memory loss, physical pain, reductions in energy and the effect of these changes on their ability to complete physical tasks like small‐scale farming, and activities of daily living important to the quality of life, such as participating in community groups. Increasing reliance on others for social, physical and financial support was also a common theme. The most prevalent health concern among participants involved the threat of non‐communicable diseases and perceptions that physical functioning may diminish.

**Conclusions:**

In rural Uganda, we found that PWH consider themselves to be healthy and do not anticipate a different ageing experience from PWOH. Common priorities shared by both groups included the desire for physical and financial independence, health maintenance and social support for daily functioning and social needs. Entities supporting geriatric care in Uganda would benefit from attention to concerns about functional limitations and reported needs as people age with and without HIV.

## 1 INTRODUCTION

Sub‐Saharan Africa (SSA) as a region is experiencing significant demographic shifts [1]. Compared to other regions of the world, SSA has a smaller proportion of older aged persons as a share of the total population [2]. However, SSA is experiencing greater increases in life expectancies than other regions, resulting in a substantial projected increase in people over 50 years old. For example, as of 2020, Uganda has approximately 3.3 million people over the age of 50 with that number projected to grow to 12.4 million by 2050 (an increase from 7% to 14% of the population [3]). This growing demographic necessitates a comprehensive understanding of the lived experiences and priorities of older people.

Nonetheless, there is a relative paucity in the literature about ageing in SSA, despite repeated calls for a better understanding of this population group [2, 4, 5, 6, 7, 8]. Data collection and research is a core element of the *WHO's Global Strategy and Action Plan for Ageing and Health* [9]. There has been notable progress in quantitative data collection to describe ageing in SSA, such as the World Health Organization Study of Global Ageing and Adult Health (SAGE) [10], Ibadan Study of Ageing in Nigeria [11] and Health and Ageing in Africa: A longitudinal study of an INDEPTH community in South Africa (HAALSI) [12]. Yet, there continues to be a need for more varied research and national and sub‐national studies. Specifically, qualitative research [13, 14, 15, 16] can provide important insights into the lived experiences of older people as they are affected by their intrinsic capacity (cognition, mobility, psychological health, vitality and sensory) and external environment as defined by the *WHO Model of Healthy Ageing* [17].

A contributing factor to the health and wellbeing of older‐age people in Uganda, and older‐age people in Africa more broadly, is the increasing availability of treatment for HIV. As HIV disease is increasingly well‐managed through antiretroviral therapy (ART), people with HIV (PWH) are living longer [4, 18, 19, 20]. As of 2019, close to 18% of the adult population with HIV in Uganda was over 50 years of age (230,000 out of 1.3 million adults with HIV) [21]. Not only are more Ugandans living longer, but many of them face the biomedical, social and emotional consequences of lifelong HIV infection.

In the global north, data on ageing PWH suggest they face an increased risk of physical comorbidities, such as cardiovascular disease [22], liver, kidney and bone disease [23], as well as premature geriatric syndromes [24, 25] compared to people without HIV. Moreover, they also must confront the impact of persistent stigma, discrimination, access to healthcare and changes in social support that may be unique to their status [26, 27, 28]. However, if and how these influences affect PWH in SSA is less well explored. Preliminary data from the region seem to suggest that PWH might have improved access to and engagement with the healthcare system [16, 29, 30] but that persistent stigma remains a major threat [31, 32, 33, 34, 35, 36]. In this qualitative study, we intended to understand the lived experiences of ageing people in rural Uganda living with and without HIV, to better understand their fears and health priorities as they grow older, and advise on the design of future research and interventions to address the needs of this population.

## 2 METHODS

The Quality of Life and Ageing with HIV in Rural Uganda study (NIH R01AG059504) is a mixed methods research study designed to identify intervention targets to improve functioning and quality of life for older aged PWH in the region. This paper describes the qualitative component of the study to understand determinants of quality of life among older aged PWH in Uganda. The results of these interviews informed the design and selection of measures for quantitative data collection for a prospective cohort study which has since begun enrolment and is ongoing. The study received approval from both the ethics committee at Mbarara University of Science and Technology, the Uganda Council for Science and Technology, as well as Mass General Brigham. Written informed consent was obtained for all participants.

Participants were consecutively sampled from an existing cohort of older individuals (48 years and older) with and without HIV [37] using two methods. First, PWH were eligible for enrolment if they had been taking ART for a minimum of 3 years and were in active care at the HIV clinic at Mbarara Regional Referral Hospital. Age and sex‐similar people without HIV (PWOH) were identified using a study‐based census of villages in the clinic catchment area and recruited from their homes [38]. We used the population study to identify comparators without HIV to minimize bias by co‐morbidity or health‐seeking behaviour that might arise from recruitment. HIV status was ascertained through annual HIV testing in the parent study and verbal confirmation of testing results included in prior study data, reflecting their lived experience. Of those approached for participation in the study, 100% agreed to participate.

In‐depth interviews were conducted by a trained interviewer using a structured interview guide. Interview guide topics were identified and selected through a literature review process in collaboration with Ugandan colleagues. Topics included (1) definitions of and priorities for quality of life in older age; (2) physical functioning, including barriers and facilitators to completing daily activities; (3) social functioning, including familial responsibilities and position in the household; (4) HIV‐related stigma; and (5) impact of multimorbidity on health and independence (see Table S1). Questions specific to the experience of living with HIV were only asked of participants with HIV. The interview guide was reassessed after it was piloted and reformatted to ensure more open‐ended questioning. Interviews took place between October 2019 and February 2020. Interviews continued until saturation was achieved. A total of 36 interviews were completed.

Interviews were conducted in Runyankole, the dominant local language, by a man in his 30s, audio‐recorded and translated to English during the transcription process. Inductive analysis was conducted using a thematic approach. A priori themes were used in the initial analysis, but additional themes and subthemes were identified as interviews were compared. Interviews were coded using Dedoose software [39]. A sample of interviews was coded by two researchers to ensure inter‐coder reliability. There was generally good agreement between coders, with any differences resolved through discussion to reach a consensus. Following the initial coding, a matrix was developed to reorganize data into headings and sub‐headings for further interpretation (see Table S2). The data were specifically analysed to examine for differences and similarities by HIV status.

## 3 RESULTS

### 3.1 Sample characteristics

The 36 study participants were equally divided by HIV serostatus and sex (Table 1). The median age of the cohort was 57 (range, 49–73). A majority of study participants reported their highest level of education to be incomplete primary school (*n* = 22, 61%). All participants reported being employed, with farming being the predominant source of livelihood among the cohort (*n* = 27, 75%). More PWOH identified as farmers compared to people with HIV (PWH) (94% vs. 56%). Additionally, the majority of the cohort reported not having a regular salary (*n* = 30, 88%), which was more common among those participants not living with HIV compared to PWH (83% vs. 94%).

**Table 1:** Cohort characteristics

	Total cohort (*N* = 36)	People with HIV (*n* = 18)	People without HIV (*n* = 18)
Age, years, range, mean	57 [49–73]	57 [49–72,62]	57 [49–73,63]
Female sex, %, *n*	18 (50)	9 (50)	9 (50)
Education			
Did not complete primary school, %, *n*	22 (61)	11 (61)	11 (61)
Completed primary school, %, *n*	12 (33)	6 (33)	6 (33)
Completed secondary school, %, *n*	2 (6)	1 (6)	1 (6)
Employed	36 (100)	18 (100)	18 (100)
Farmer, %, *n*	27 (75)	10 (56)	17 (94)
No regular salary,^a^ %, *n*	30 (88)	15 (83)	15 (94)
Marital status			
Single, %, *n*	1 (3)	1 (6)	0 (0)
Married/cohabitating, %, *n*	23 (64)	10 (56)	13 (72)
Divorced/separated, %, *n*	2 (6)	1 (6)	1 (6)
Widowed, %, *n*	10 (28)	6 (33)	4 (22)
Family structure			
Living alone, %, *n*	3 (8)	3 (17)	0 (0)
Has biological children,^b^ %, *n*	34 (94)	17 (94)	17 (94)
Current primary caretaker for non‐biological children,^c^ %, *n*	20 (56)	10 (56)	10 (56)

^a^ Two HIV‐negative participants missing data, *n* = 34.

^b^ Includes grown children who have moved out of the house.

^c^ Includes nieces, nephews, and other relatives.

Approximately two‐thirds of the study participants were married or cohabitating (*n* = 23, 64%). Only three (8%) of participants lived alone (17% [*n* = 3] among PWH vs. none of PWOH). Most participants have living biological children (*n* = 34, 94%) and half are the current primary caretaker for additional non‐biological children (*n* = 20, 56%).

### 3.2 Self‐perceived impact of HIV on health and quality of life

Generally, PWH did not consider their health or quality of life to be dramatically different from PWOH as they age. In fact, many PWH considered themselves healthy, their HIV status notwithstanding. “[HIV] does not affect me at any time and I might be better than HIV negative people,” said a 57‐year‐old woman with HIV, a common sentiment. Another female respondent of 66 years noted her similarity to PWOH when she said, “Anyone can fall sick of anything whether they have HIV or not. There are people that are prone to any sicknesses regardless of having HIV or not.”

Respondents did note there is a difference between those with well‐controlled disease and those without the same level of adherence. One 72‐year‐old woman with HIV described it in the following way: “HIV is like flu or cough; if you are taking your medication as prescribed by the doctor, it does not affect your way of doing things in any way. [Sickness] happens to those who don't take their medication very well.” One 70‐year‐old man illustrated a common sentiment among the participants about their expected longevity with ART:

I am now confident that I will live for many years until the true medicine is discovered. I am also confident that when I continue taking my medication, I will still live and serve my family, the church, and the community as long as I am still taking my medication and not disorganized with old age.

Nonetheless, for a minority of participants, the knowledge of their HIV serostatus continues to affect the way they see themselves and their overall perception of their health. “Because of taking the drug daily, you feel no self‐love, and if you happen to become sick by something small, you'll get worried that other sicknesses will come such as TB,” said one 55‐year‐old woman with HIV.

### 3.3 Changing perceptions about HIV in the ART era

Both PWH and PWOH noted historical shifts in the way HIV impacted families and individuals. PWH highlighted the burden of stigma and discrimination early in their disease progression prior to freely available treatment. “I'd […] ask them when they'd visit me, but they'd say they will make it. So, I'd know if it's due to the virus that they can't show up. Afterwards, all that ended, and they understood how HIV is spread. They'd eat rice with me,” said a 66‐year‐old woman with HIV.

Respondents frequently told of how life has improved for both themselves and their communities with better treatment and knowledge of the virus. As one 49‐year‐old man with HIV put it, “Honestly speaking, there is no HIV person that's treated unfairly! There is no trademark that points out an HIV positive person. Before they were affected by skin rash, and everyone knew the signs. Can you tell that I am HIV by merely looking at my skin?” While there was limited reporting of maltreatment of PWH, it was usually rationalized with some qualifier such as the person did not take ART routinely, was promiscuous or was self‐stigmatizing. PWOH tended to agree with their PWH counterparts when they say, “now a person living with HIV is treated like any normal person […] He will remain healthy and will suffer other disease like any other people in the community” (57‐year‐old woman without HIV).

The most commonly cited reasons for this change in treatment and perceptions about PWH were the increasing availability of treatment for HIV and the pervasiveness of HIV infection in the community. As one 51‐year‐old woman without HIV stated, PWH are not stigmatized, “because one is worried that tomorrow or another day he will be HIV positive also.” Most respondents noted this shift was most evident soon after ART became widely available for free.

### 3.4 Shared experiences of physical and mental declines with ageing

PWH are not immune to the effects of ageing and felt that they are ageing similarly to their peers. A 49‐year‐old man with HIV sums it up when he says, I view myself as a normal person and I would not see any change of having HIV because I don't see any difference; I have developments just like an HIV‐negative person only that I see that I don't have the energy that I had before like now.

While facing the expected declines of ageing, most HIV‐positive respondents consider themselves to be healthy. By contrast, most PWOH generally considered themselves to have poor health due to ageing. One man without HIV of 52 years described his current health as the following: My hands pain, the whole body pains. That means that old age is affecting me. Because, back then I would work and then feel okay afterwards. But now, that is not the case. I feel all joints hardening and paining and I fail to work as I used to.

These health complaints are not unique to PWOH. When asked about health changes as they have aged, participants across all sub‐groups reported reduced energy and strength, increased physical pains and aches, changes in sleep patterns and memory loss.

My health has changed because nowadays I usually feel constant headache, sometimes I feel dizziness, my eyes are not seeing properly, and when I stand for a long time, I find myself in a fatigued state. When I work for a long time and I force myself to go an extra mile, I become very tired to the extent that when I sit somewhere I feel I do not want to move away until I have rested enough (52‐year‐old woman without HIV).

While some respondents attribute their declining functionality to a particular health condition (including HIV among PWH), most believe it is part of the normal ageing process. “When I go to the health facility and they don't diagnose any disease, then that makes me think that it is due to ageing,” said a 59‐year‐old man without HIV.

Another concern for most respondents is changes in memory. Apart from some younger participants (under 60 years), respondents noted increased memory loss ranging from benign complaints, such as forgetting where they put their phone, to concerns of the impact on their daily activities. Respondents attributed these changes to multiple different causes: normal ageing, having “too many thoughts” (a synonym for worrying) and responsibilities, and, for PWH, a theory that it was due to a combination of ageing and HIV. One 62‐year‐old man with HIV alluded to such: “I really feel someone of 62 years wouldn't have started becoming forgetful.” A number of respondents in both groups felt that with ageing comes more stressors and responsibility that affects memory and mental health. For example, a 55‐year‐old woman with HIV felt this way when she said:

Those are people who will have had a lot of things to think about as they grow, for instance when a family is not independent as regards their needs, that's when you will find the head of the household thinking about a lot, thus in their old age they start to lose their memory.

While respondents did not believe memory loss could be treated, they believe it could be prevented or improved through reading, learning and maintaining relationships.

The stress that can cause memory loss was also reported to affect mood. Many respondents noted periods of depression, despondency and anxiety often brought about by situational stressors, such as fear of illness and death, financial constraints and lack of basic necessities for themselves and family. For example, one woman with HIV of 62 years said, “But for me being HIV positive I am always thinking I might die anytime.” Notably, health concerns were less frequently cited as causes of depression or anxiety as compared to social stressors.

### 3.5 Concerns about functional declines in daily activities with age

Most respondents highlighted shifts in their routine activities as they have aged. Some examples of limitations include reading less due to changing eyesight, walking and biking shorter distances or diminished socializing and participating in community groups. One woman without HIV of 57 years said she would “fail to maintain my position at the [Local Council] committee because I can no longer write and so I will not disturb anyone to keep writing for me.” Loss of sexual desire was also mentioned by both groups. As one 64‐year‐old man with HIV said, “Back in the days, we young men would not bypass ‘a dress’, but these days I look at women like a dog would be looking at money notes.” Even more disruptive is the decreasing ability to “dig” (farm and conduct small‐scale food provision), including gardening, grazing of animals, selling of produce, and construction work—the means of income for many respondents. “I used to remove the banana suckers myself but now I cannot. I would like to slash my compound, but I cannot manage it now, and now I have to use money to hire someone to do it. Now even raising a hoe is becoming a challenge and I may soon fail to dig,” said a 54‐year‐old woman living with HIV.

Recognition that decreases in energy and increased chronic pain affect daily activities was nearly universal among respondents. Younger participants did not report major changes in their usual activities, but they anticipate changes as they continue to grow older. Among PWH, there is uncertainty if pain and loss of energy are associated with their HIV infection or ageing. Some seem quite certain that their physical changes are attributable to HIV infection or its treatment such as a 62‐year‐old male PWH who insists “Way back when I had not acquired HIV, I used to do all my work myself because I used to be energetic, unlike nowadays when I feel I am no longer so strong to accomplish all my activities.” Other PWH respondents are more equivocal, such as a 55‐year‐old respondent who exclaimed, “I try to work sometimes and feel pain so I ask myself if its old age or it's HIV epidemic!” while others are insistent that HIV infection plays no role in their changing activities, such as a 66‐year‐old woman who reported, “I see old age in my life, because when I look back on my life and how I used to do a lot of chores with a child on back whilst fetching firewood; which I now walk the same distance but feel tired and notice that it's due to old age and not the HIV epidemic.” Apart from HIV infection, respondents identify the cause of their challenges to usual activities to include reduction in energy and stamina, pain and physical weakness, other illnesses, increased sleep, and changes in memory and mental reasoning.

To accommodate changes in physical and mental ability, many respondents receive assistance with their usual activities. The primary activities which are assisted include working in the garden or plantation (weeding and harvesting), collecting and splitting firewood, cooking, cleaning, fetching water and grazing animals. Most often they described family members or younger people living with the respondents who serve this role, but occasionally it is friends, neighbours or hired help. One 73‐year‐old woman without HIV said, “I used to carry a full jerrycan of water but now I even struggle carrying a small jerrycan of water and that is why I stay with some children so that they can help me in one way or another.” Both PWH and PWOH worry that some activities may go neglected or change as help diminishes, and they do not have the funds to hire casual workers.

### 3.6 Health fears for the future

When participants were asked what conditions they are worried about developing in the future, many participants reported their primary concerns being developing uncurable, non‐communicable diseases, such as cancer, diabetes and hypertension, with a particular emphasis on cancer. A 57‐year‐old woman without HIV sums it up when she says:

Cancer […] claims lives of many people and you hear someone saying that instead of being killed by cancer I'd rather be killed by HIV. One says that with HIV, he or she can access any health facility and get tested and treatment accordingly and become fine. But with cancer, one has to die while in terrible pain.

Even among PWH, respondents fear non‐communicable diseases. “[I] am worried about diabetes, blood pressure, and cancer. If those affect me then I won't fair well,” said a 53‐year‐old man with HIV.

Respondents were also concerned about changes in physical abilities that would negatively impact social and physical functioning, such as deteriorating eyesight, hearing loss and loss of use of their hands or legs. A 49‐year‐old man with HIV said, “I ask God to help me not to ever break any of my limbs especially the arms because it'd be bad for me and my family, or what if I lost my eye, what would happen to me!” Respondents have very real fears of declining health and their ability to maintain their current lifestyle. For example, a 62‐year‐old man with HIV says, “I have a feeling of how my energy to do work keeps reducing. For example, yesterday I worked in my banana plantation, but that alone left me so weak through the whole night. This is a clear sign that my health is really deteriorating.”

Lastly, respondents worry about acquiring infectious diseases, including tuberculosis and HIV (among the PWOH). “I fear getting infected with HIV because that virus deteriorates someone's health completely, and so there are some activities you will no longer do when you have HIV, which in turn reduces on the household income, as more money goes to treatment,” said a 56‐year‐old woman without HIV. Apart from the fear of developing HIV, these responses were largely consistent across sex and HIV serostatus.

### 3.7 Priorities for the future as they age

When asked about ideal ageing and priorities for themselves as they age, the responses aligned across gender, age and HIV status. According to a 60‐year‐old woman without HIV, ideal ageing looks like someone who:

has what to eat and drink, has a good house to stay in, and she or he has prepared where to get some income like having livestock at home, and food security at home and he even has a motorcycle to take him to the hospital in case of sickness and does not depend on other people to survive or beg other people to give him or her money to do something.

The common themes coalesced around adequate nutrition, help with physical tasks, income‐generating sources, such as land and livestock, a respectable home and transportation.

Financial security was seen by most as the key factor that allows for other goals and necessities to be met. It prevents dependency and ensures access to medical care. “The most poisonous situation is to become a needy person at an older age. For example, when one cannot even afford to buy himself a ‘Headex’ pill,” said one HIV‐positive 64‐year‐old man. Particularly, more passive income is most appealing as this group ages, whether that is through plantations with hired help, grazing livestock, rental properties or business that others work.

A repeated priority for ageing is having access to routine care and company around the house whether that is hired or family, with the preference being children or grandchildren.

When I grow older than now, I would wish to be seated in one place, playing and having fun with my grandchildren as they make me smile, make fun of me, like how I used to play and make fun of my grandparents when I was still young, and I don't want to keep alone because I feel I will get bored if I keep alone […] I want my children to be near me so that I am able to tell them or ask them whatever I want and they give it to me in time. For instance if I tell them that I want like bread or rice, I need them to give it to me, because when they are far and I fail to get it, I may not feel happy (72‐year‐old HIV‐positive woman).

Good health is something respondents aspire to maintain but recognize they may not be able to control. “Above all, good health is my priority because I want to have good health. Because if you have good health, others can follow,” said a 59‐year‐old man without HIV. Part of good health is defined as being “peaceful” among respondents, which can be described as living without stress. A woman of 60 without HIV said, “I need to be with good health, free of diseases and also to be peaceful.” Among some HIV‐positive respondents, managing their HIV by adhering to their treatment regimen is a priority as they age, but it was not a commonly reported concern.

## 4 DISCUSSION

This study investigated the experiences of older aged Ugandans, both with HIV and without, related to the quality of life, daily functioning and long‐term health. Our findings suggest the differences between PWH and PWOH are subtle. In most cases, perceived quality of life and health was greater among PWH than among PWOH in our study. Intrinsic capacity (cognition, mobility, psychological health, vitality and sensory) [17] between the two groups seems similar, while both groups face changing physical functioning, activity levels, memory and disease burden as they continue to age.

A potentially unexpected finding from this work was the relative similarity in self‐perceptions about health, quality of life, independence and longevity between people with and without HIV. By and large, this differs from work in the global north and elsewhere that has explored experiences of living with HIV [40, 41, 42, 43] but corroborates findings from other research in Uganda [16]. The relative similarity we found between the groups could potentially be attributed to the study setting: namely, an environment where ART is largely available (particularly to those who we interviewed who, by design, were linked to HIV care) and where HIV stigma has decreased somewhat [31] despite its persistence in other domains [13, 32, 44]. There was recognition of the devastating impact of HIV in the past, but fewer expressed concerns about its impact on individuals and the community more broadly at present, even among PWH. While topics of self‐perceived stigma among PWH were not specifically addressed in these interviews, our findings correlate with other research that has shown a greater perceived quality of life and resilience [45] with less internalized stigma among PWH [46]. This finding may also reflect a PWH population that is engaged in care and has a well‐controlled disease [47, 48] rather than those facing a new HIV diagnosis or delaying treatment [32, 49, 50]. It may also suggest that self‐perceived health can differ significantly from clinically defined health [51].

Our data suggest that health concerns, including among PWH, are principally related to non‐communicable diseases and health conditions that could affect older‐aged persons’ ability to work and socialize. Notably, we found little evidence that older PWH are specifically concerned about the effects of HIV on their health or longevity. These health concerns follow worldwide trends in health threats evolving from infectious diseases to non‐communicable diseases, including for PWH [52]. Pain, reduced energy and memory loss all have an impact on daily activities among older‐aged Ugandans, with reports of pain and reductions in energy being nearly universal. These findings are similar to the existing evidence identifying the loss of independence in day‐to‐day functioning as a primary concern for older‐aged persons [51, 53].

As people age, they have real concerns over financial stability, accessibility of medical care, food security, assistance with daily needs by caregivers and social contact. With a nascent geriatric care programme in Africa [54], there is the opportunity to design programmes to effectively meet the needs as well as the priorities of the ageing population. From a health perspective, there is a need for mental health support, pain management, routine evaluations for causes of fatigue, palliative care for chronic diseases and independent living skills programmes, all of which need to be affordable and accessible [9, 55]. From a financial and food security perspective, there is room for programmes, such as community savings groups (which are already in use), extension work to support farmers in their later years, business development programmes for older aged Africans and social security safety nets for the most disadvantaged. For social contact and assistance, social support groups and home care programmes can be implemented to support the social and cognitive functioning of the ageing population as well as the mental health and social isolation of family caregivers [56, 57]. The ageing population in SSA is quickly growing, and these programmes and policies are urgently needed.

Our findings should be interpreted in light of several limitations. There are some inherent differences between the PWH respondents and their PWOH peers. PWH on ART and in clinical care may reflect a population better engaged in healthcare and should not be taken to represent the experiences of those not on treatment. Additionally, the PWOH in this study were recruited from a slightly more rural area compared to the PWH respondents [58], but the two groups were approximately similar in as many ways as we could control. It is possible that HIV co‐morbidities and illnesses apart from HIV that are not quantified may have influenced responses. The interviews were translated and transcribed before being analysed, and some nuance may have been lost in the process despite using native speakers for both interviewing and transcribing. The participants were drawn from a cohort with the non‐standard age range of 48 and older, making comparisons to studies that utilize 50 years and older more difficult. Lastly, while not a limitation per se, qualitative research is not meant to be representative of all PWH and the corresponding population without HIV. Findings are, therefore, not generalizable to the entire population.

## 5 CONCLUSIONS

With changing demographic structures in the era of the widespread availability of HIV treatment, it will be critical to understand the priority and concerns of older‐aged individuals as they represent an increasing proportion of this key population. In rural Uganda, we found that concerns of ageing are nearly universal, irrespective of HIV status, and include fears about acquiring non‐communicable diseases, loss of independent physical functioning and diminished energy, as well as memory loss. Priorities for the future were financial and physical independence, access to care when needed, health maintenance and social connection. Medical care [9] and policy [55] should be designed to meet the needs of this growing population in SSA to ensure healthy ageing.

## COMPETING INTERESTS

ACT reports receiving a financial honorarium from Elsevier, Inc. for his work as Co‐Editor in Chief of the Elsevier‐owned journal *SSM‐Mental Health*. All other authors declare no competing interests.

## AUTHORS’ CONTRIBUTIONS

ZR and RG conceptualized and drafted the manuscript as well as conducted the analysis. RS oversaw in‐country data collection. MJS designed the research study. ACM, DS, SO, NN, MG, JS, ACT and SA were involved in drafting the manuscript and provided critical feedback on the full manuscript. All authors read and approved the final manuscript.

## FUNDING

The Quality of Life and Ageing with HIV in Rural Uganda is funded by the U.S. National Institute on Ageing (R01AG059504). Additional funding was provided by the U.S. National Institute of Mental Health (R01MH113494).

## Supporting information


**Table S1**. Examples of interview questions by domain.
**Table S2**. Illustrative quotes by theme.

## Data Availability

The data that support the findings of this study are available from the corresponding author upon reasonable request.

## References

[1] Velkoff VA , Kowal PR . Aging in sub‐Saharan Africa: the changing demography of the region. National Academies Press; 2006.

[2] Aboderin IAG , Beard JR . Older people's health in sub‐Saharan Africa. Lancet. 2015;385(9968):e9–11. 25468150 10.1016/S0140-6736(14)61602-0

[3] United National Department of Population and Economic Affairs . Population Division World Population Prospects 2019, Data Query [Internet]. 2020 [cited 2020 Dec 14]. Available from: https://population.un.org/wpp/DataQuery/

[4] Negin J , Bärnighausen T , Lundgren JD , Mills EJ . Aging with HIV in Africa: the challenges of living longer. AIDS. 2012;26:S1–5. 22713477 10.1097/QAD.0b013e3283560f54 PMC4017661

[5] Effros RB , Fletcher CV , Gebo K , Halter JB , Hazzard WR , Horne FM , et al. Aging and infectious diseases: workshop on HIV infection and aging: what is known and future research directions. Clin Infect Dis. 2008;47(4):542–53. 18627268 10.1086/590150 PMC3130308

[6] Aboderin I . Coming into its own? Developments and challenges for research on aging in Africa. J Gerontol B Psychol Sci Soc Sci. 2017;72(4):643–5. 27038399 10.1093/geronb/gbw017

[7] Kowal P , Peachey K . Indicators for the minimum data set project on ageing: a critical review in sub‐Saharan Africa. Dar es Salaam, United Republic of Tanzania: World Health Organization, HelpAge International, U.S. National Institute on Aging; 2001.

[8] Harris TG , Rabkin M , El‐Sadr WM . Achieving the fourth 90: healthy aging for people living with HIV. AIDS. 2018;32(12):1563–9. 29762172 10.1097/QAD.0000000000001870 PMC6082594

[9] Rudnicka E , Napierała P , Podfigurna A , Męczekalski B , Smolarczyk R , Grymowicz M . The World Health Organization (WHO) approach to healthy ageing. Maturitas. 2020;139:6–11. 32747042 10.1016/j.maturitas.2020.05.018 PMC7250103

[10] Chatterji S . World Health Organisation's (WHO) Study on Global Ageing and Adult Health (SAGE). BMC Proc. 2013; 7:S1. 10.1186/1753-6561-7-S4-S1 PMC3892776 24764490

[11] Gureje O , Kola L , Afolabi E , Olley BO . Determinants of quality of life of elderly Nigerians: results from the Ibadan study of ageing. Afr J Med Med Sci. 2008;37(3):239–47. 18982816 PMC2820711

[12] Gómez‐Olivé FX , Montana L , Wagner RG , Kabudula CW , Rohr JK , Kahn K , et al. Cohort Profile: Health and Ageing in Africa: a Longitudinal Study of an INDEPTH Community in South Africa (HAALSI). Int J Epidemiol. 2018;47(3):689–90j. 29325152 10.1093/ije/dyx247 PMC6005147

[13] Kuteesa MO , Seeley J , Cumming RG , Negin J . Older people living with HIV in Uganda: understanding their experience and needs. Afr J AIDS Res. 2012;11(4):295–305. 25860188 10.2989/16085906.2012.754829

[14] Schatz E , Seeley J , Negin J , Mugisha J . They ‘don't cure old age’: older Ugandans’ delays to health‐care access. Ageing Soc. 2018;38(11):2197–217.

[15] Wright S , Zalwango F , Seeley J , Mugisha J , Scholten F . Despondency among HIV‐positive older men and women in Uganda. J Cross Cult Gerontol. 2012;27(4):319–33. 22930234 10.1007/s10823-012-9178-x PMC3505509

[16] Negin J , Nyirenda M , Seeley J , Mutevedzi P . Inequality in health status among older adults in Africa: the surprising impact of anti‐retroviral treatment. J Cross Cult Gerontol. 2013;28(4):491–3. 24122525 10.1007/s10823-013-9215-4

[17] Ahmad A , Neelamegam M , Rajasuriar R . Ageing with HIV: health implications and evolving care needs. J Int AIDS Soc. 2020;23(9):e25621. 32996718 10.1002/jia2.25621 PMC7526224

[18] Joint United Nations Programme on HIV/AIDS . HIV and aging. [Internet]. 2013 [cited 2020 Dec 14]. Available from: http://www.unaids.org/en/media/unaids/contentassets/documents/unaidspublication/2013/20131101_JC2563_hiv‐and‐aging_en.pdf

[19] Hontelez JAC , de Vlas SJ , Baltussen R , Newell ML , Bakker R , Tanser F , et al. The impact of antiretroviral treatment on the age composition of the HIV epidemic in sub‐Saharan Africa. AIDS. 2012;26(Suppl 1):S19–30. 22781175 10.1097/QAD.0b013e3283558526 PMC3886374

[20] Bor J , Herbst AJ , Newell ML , Barnighausen T . Increases in adult life expectancy in rural South Africa: valuing the scale‐up of HIV treatment. Science. 2013;339(6122):961–5. 23430655 10.1126/science.1230413 PMC3860268

[21] UNAIDS . AIDSinfo “People living with HIV receiving ART” [Internet]. AIDSinfo. 2020 [cited 2020 Dec 14]. Available from: https://aidsinfo.unaids.org/

[22] Okello S , Amir A , Bloomfield GS , Kentoffio K , Lugobe HM , Reynolds Z , et al. Prevention of cardiovascular disease among people living with HIV in sub‐Saharan Africa. Prog Cardiovasc Dis. 2020;63(2):149–59. 32035126 10.1016/j.pcad.2020.02.004 PMC7237320

[23] Deeks SG , Tracy R , Douek DC . Systemic effects of inflammation on health during chronic HIV infection. Immunity. 2013;39(4):633–45. 24138880 10.1016/j.immuni.2013.10.001 PMC4012895

[24] Greene M , Covinsky KE , Valcour V , Miao Y , Madamba J , Lampiris H , et al. Geriatric syndromes in older HIV‐infected adults. J Acquir Immune Defic Syndr. 2015;69(2):161–7. 26009828 10.1097/QAI.0000000000000556 PMC4445476

[25] Rodriguez‐Penney AT , Iudicello JE , Riggs PK , Doyle K , Ellis RJ , Letendre SL , et al. Co‐morbidities in persons infected with HIV: increased burden with older age and negative effects on health‐related quality of life. AIDS Patient Care STDs. 2013;27(1):5–16. 23305257 10.1089/apc.2012.0329 PMC3545369

[26] The MATCH Study Group , Neuman M , Obermeyer CM . Experiences of stigma, discrimination, care and support among people living with HIV: a four country study. AIDS Behav. 2013;17(5):1796–808. 23479002 10.1007/s10461-013-0432-1 PMC3671197

[27] Siegler EL , Brennan‐Ing M . Adapting systems of care for people aging with HIV. J Assoc Nurses AIDS Care. 2017;28(5):698–707. 28602461 10.1016/j.jana.2017.05.006

[28] Roger KS , Mignone J , Kirkland S . Social aspects of HIV/AIDS and aging: a thematic review. Can J Aging. 2013;32(3):298–306. 23941598 10.1017/S0714980813000330

[29] Manne‐Goehler J , Montana L , Gómez‐Olivé FX , Rohr J , Harling G , Wagner RG , et al. The ART advantage: health care utilization for diabetes and hypertension in rural South Africa. J Acquir Immune Defic Syndr. 2017;75(5):561–7. 28696346 10.1097/QAI.0000000000001445 PMC5516957

[30] Kwarisiima D , Atukunda M , Owaraganise A , Chamie G , Clark T , Kabami J , et al. Hypertension control in integrated HIV and chronic disease clinics in Uganda in the SEARCH study. BMC Public Health. 2019;19(1):511. 31060545 10.1186/s12889-019-6838-6 PMC6501396

[31] Chan BT , Tsai AC , Siedner MJ . HIV treatment scale‐up and HIV‐related stigma in sub‐Saharan Africa: a longitudinal cross‐country analysis. Am J Public Health. 2015;105(8):1581–7. 26066939 10.2105/AJPH.2015.302716 PMC4500171

[32] Chan BT , Weiser SD , Boum Y , Siedner MJ , Mocello AR , Haberer JE , et al. Persistent HIV‐related stigma in rural Uganda during a period of increasing HIV incidence despite treatment expansion. AIDS. 2015;29(1):83–90. 25268886 10.1097/QAD.0000000000000495 PMC4286463

[33] Akatukwasa C , Getahun M , El Ayadi AM , Namanya J , Maeri I , Itiakorit H , et al. Dimensions of HIV‐related stigma in rural communities in Kenya and Uganda at the start of a large HIV ‘test and treat’ trial. PLoS One. 2021;16(5):e0249462. 33999961 10.1371/journal.pone.0249462 PMC8128261

[34] Viljoen L , Bond VA , Reynolds LJ , Mubekapi‐Musadaidzwa C , Baloyi D , Ndubani R , et al. Universal HIV testing and treatment and HIV stigma reduction: a comparative thematic analysis of qualitative data from the HPTN 071 (PopART) trial in South Africa and Zambia. Sociol Health Illn. 2021;43(1):167–85. 33085116 10.1111/1467-9566.13208 PMC7894283

[35] Stangl AL , Pliakas T , Mainga T , Steinhaus M , Mubekapi‐Musadaidzwa C , Viljoen L , et al. The effect of universal testing and treatment on HIV stigma in 21 communities in Zambia and South Africa. AIDS. 2020;34(14):2125–35. 32773484 10.1097/QAD.0000000000002658 PMC8425632

[36] Hargreaves JR , Krishnaratne S , Mathema H , Lilleston PS , Sievwright K , Mandla N , et al. Individual and community‐level risk factors for HIV stigma in 21 Zambian and South African communities: analysis of data from the HPTN071 (PopART) study. AIDS. 2018;32(6):783–93. 29369164 10.1097/QAD.0000000000001757 PMC5854764

[37] Siedner MJ , Kim JH , Nakku RS , Bibangambah P , Hemphill L , Triant VA , et al. Persistent immune activation and carotid atherosclerosis in HIV‐infected Ugandans receiving antiretroviral therapy. J Infect Dis. 2016;213(3):370–8. 26347573 10.1093/infdis/jiv450 PMC4704672

[38] Takada S , Nyakato V , Nishi A , O'Malley AJ , Kakuhikire B , Perkins JM , et al. The social network context of HIV stigma: population‐based, sociocentric network study in rural Uganda. Soc Sci Med. 2019;233:229–36. 31229909 10.1016/j.socscimed.2019.05.012 PMC6827718

[39] Dedoose web application for managing, analyzing, and presenting qualitative and mixed method research data. Los Angeles, CA: SocioCultural Research Consultants, LLC; 2018.

[40] Kahana E , Kahana B . Successful aging among people with HIV/AIDS. J Clin Epidemiol. 2001;54(12):S53–6. 11750210 10.1016/s0895-4356(01)00447-4

[41] Balderson BH , Grothaus L , Harrison RG , McCoy K , Mahoney C , Catz S . Chronic illness burden and quality of life in an aging HIV population. AIDS Care. 2013;25(4):451–8. 22894702 10.1080/09540121.2012.712669 PMC3535557

[42] The HIV Neurobehavioral Research Program (HNRP) Group , Moore RC , Fazeli PL , Jeste DV , Moore DJ , Grant I , et al. Successful cognitive aging and health‐related quality of life in younger and older adults infected with HIV. AIDS Behav. 2014;18(6):1186–97. 24633788 10.1007/s10461-014-0743-x PMC4020963

[43] Shippy RA , Karpiak SE . Perceptions of support among older adults with HIV. Res Aging. 2005;27(3):290–306.

[44] Kuteesa MO , Wright S , Seeley J , Mugisha J , Kinyanda E , Kakembo F , et al. Experiences of HIV‐related stigma among HIV‐positive older persons in Uganda – a mixed methods analysis. SAHARA‐J. 2014;11(1):126–37. 25053275 10.1080/17290376.2014.938103 PMC4272102

[45] Gottert A , McClair TL , Pulerwitz J , Friedland BA . What shapes resilience among people living with HIV? A multi‐country analysis of data from the PLHIV Stigma Index 2.0. AIDS. 2020;34(1):S19–31. 32881791 10.1097/QAD.0000000000002587

[46] Nobre N , Pereira M , Roine RP , Sutinen J , Sintonen H . HIV‐related self‐stigma and health‐related quality of life of people living with HIV in Finland. J Assoc Nurses AIDS Care. 2018;29(2):254–65. 28935441 10.1016/j.jana.2017.08.006

[47] Bebell LM , Kembabazi A , Musinguzi N , Martin JN , Hunt PW , Boum Y , et al. Internalized stigma, depressive symptoms, and the modifying role of antiretroviral therapy: a cohort study in rural Uganda. SSM ‐ Mental Health. 2021;1:100034. 35252904 10.1016/j.ssmmh.2021.100034 PMC8896824

[48] Tsai AC , Bangsberg DR , Bwana M , Haberer JE , Frongillo EA , Muzoora C , et al. How does antiretroviral treatment attenuate the stigma of HIV? Evidence from a cohort study in rural Uganda. AIDS Behav. 2013;17(8):2725–31. 23670710 10.1007/s10461-013-0503-3 PMC3773278

[49] The HPTN 071 (PopART) Study Team , Seeley J , Bond V , Yang B , Floyd S , MacLeod D , et al. Understanding the time needed to link to care and start ART in seven HPTN 071 (PopART) study communities in Zambia and South Africa. AIDS Behav. 2019;23(4):929–46. 30415432 10.1007/s10461-018-2335-7 PMC6458981

[50] Kawuma R , Seeley J , Mupambireyi Z , Cowan F , Bernays S , REALITY Trial Team . “Treatment is not yet necessary”: delays in seeking access to HIV treatment in Uganda and Zimbabwe. Afr J AIDS Res. 2018;17(3):217–25. 30132397 10.2989/16085906.2018.1490785

[51] Tkatch R , Musich S , MacLeod S , Kraemer S , Hawkins K , Wicker ER , et al. A qualitative study to examine older adults’ perceptions of health: keys to aging successfully. Geriatr Nurs. 2017;38(6):485–90. 28341064 10.1016/j.gerinurse.2017.02.009

[52] Fallon CK , Karlawish J . Is the WHO definition of health aging well? Frameworks for “health” after three score and ten. Am J Public Health. 2019;109(8):1104–6. 31268759 10.2105/AJPH.2019.305177 PMC6611105

[53] Sabin CA , Harding R , Bagkeris E , Nkhoma K , Post FA , Sachikonye M , et al. Pain in people living with HIV and its association with healthcare resource use, well being and functional status. AIDS. 2018;32(18):2697–706. 30289809 10.1097/QAD.0000000000002021

[54] Apt NA . Aging in Africa: past experiences and strategic directions. Ageing Int. 2012;37(1):93–103.

[55] Saka S , Oosthuizen F , Nlooto M . National policies and older people's healthcare in sub‐Saharan Africa: a scoping review. Ann Glob Health. 2019;85(1):91. 31251482 10.5334/aogh.2401 PMC6634477

[56] Ainamani HE , Alele PE , Rukundo GZ , Maling S , Wakida EK , Obua C , et al. Caregiving burden and mental health problems among family caregivers of people with dementia in rural Uganda. Glob Ment Health. 2020;7:e13. 10.1017/gmh.2020.7 PMC7379317 32742671

[57] Ainamani HE , Alele PE , Rukundo GZ , Maling S , Wakida EK , Obua C , et al. Caring for people with dementia in rural Uganda: qualitative study of caregiving burden experienced by informal and formal caregivers. J Glob Health Rep. 2020;4:e2020038. 33043153 10.29392/001c.12848 PMC7544160

[58] van der Hoeven M , Kruger A , Greeff M . Differences in health care seeking behaviour between rural and urban communities in South Africa. Int J Equity Health. 2012;11(1):31. 22691443 10.1186/1475-9276-11-31 PMC3419677

