# Supplementary material for: Priorities for health and wellbeing for older people with and without HIV in Uganda: a qualitative methods study
Source: J Int AIDS Soc. 2022 Sep 29;25(Suppl 4):e26000. doi: 10.1002/jia2.26000 (PMC9523001; doi:10.1002/jia2.26000)
Supplement: Supplementary file 1 — Table S1. Examples of interview questions by domain. Table S2. Illustrative quotes by theme. [file JIA2-25-e26000-s001.docx]

**Supplemental Materials**

| **Supplemental Table 1. Examples of interview questions by domain** | |
| --- | --- |
| **Domain** | **Examples of questions** |
| Quality of life with aging | In your community, who are the elderly people that you think are living a good life? What makes their life good? |
| Physical functioning and self-care | What challenges have you found to completing your usual activities? |
| Multimorbidity | What change in your health as you have gotten older has had the largest impact on your life? How has it affected your life? |
| Economic security | How has your ability to contribute to your household’s economic well-being changed as you have gotten older? |
| Care giving | Other than financial support, what types of things do your family or friends rely on you for? |
| Social support | How do you feel your age has impacted your standing in the community? |
| HIV- and age-related stigma | Can you tell me about a time that you felt you were treated differently because of your age? |

| **Supplemental Table 2. Illustrative quotes by theme** | |  |
| --- | --- | --- |
| **Theme** | **Quotes** |  |
|  | People with HIV | HIV negative |
| Controlled HIV does not have a major impact on health | If you follow the medical advice, prescriptions, eating healthy then you can live more years with HIV/AIDS. - Male, 60  For instance, I do digging/cultivation very well, someone might not believe that I am a person living with HIV. - Female, 55 | There is a change in a sense that once a person tests and discover HIV early, and goes and enroll on ARVs early, one will live a healthier life. He will remain healthy and will suffer other disease like any other people in the community. - Female, 57 |
| HIV contributes to health fears for PWH | Much as they say that once you take the drugs as prescribed, then you don’t become affected by the casual sicknesses, but you cannot be the same as an HIV negative person. - Male, 49  Such HIV negative people and who are not suffering from NCDs have no worries on their health, for me every time I feel weak, I always think of HIV. - Female, 60 | N/A |
| Availability of antiretroviral therapy improves perceptions of PWH | Whoever had HIV that time would be considered as a dead one but now, whoever has it is considered to be in the same boat with others that are sick of other sicknesses. […] So the drugs have really helped much. - Female, 53 | Now people treat them well and HIV positive persons are able to live long because of the availability of ARVs and now they are treated well by their relatives and community members. - Female, 49 |
| Pervasiveness of HIV infection reduces stigma | How will they gossip about me when their own father is sick too or either have about two or three person that are HIV positive in the family? So how will you talk about me? Since I pass where you pass too. - Male, 49 | There is no need of telling them that you are negative because even those who do not have it will get it. - Female, 60 |
| PWH have better perceived health | I really do not find a difference, or I would even say I am better-off than them, because there are people who are so weak that they can’t even manage to go to the garden when actually they are HIV negative. - Female, 55 | A person with good health is a person free from diseases like cough, ability to fight against flew and other opportunistic infections. And in my youthful days I rarely suffered from flew or fever but nowadays I develop headache now and then and I am gradually growing weaker. - Male, 65 |
| Memory loss is a common sign of ageing | It is brought about by age because the older you grow the more your memory goes degenerating. - Male, 70  When I chat with a person of my age, she will mention that she forgets too but it’s different since she has no HIV. When I compare how much she forgets by what she tells me, I see that I actually forget more than her, while we are the same but is see that mine is all mixed with HIV epidemic. - Female, 53 | I attribute [memory loss] to aging because before I became aged, whenever I set out to collect a cup or any other item, I would straight away get it. But nowadays, I easily forget and start asking myself what I had gone to collect. - Male, 50 |
| Stress affects mood | Bad thoughts are caused by money, food and people that disturb. - Male, 60 | Even now my emotions are affected because I have not completed school fees for my children so even when I am there chatting with my friends I found myself already diverted by thinking of where I will get the remaining school fees for my children. So such thoughts affect my emotions. - Female, 49 |
| Decreased energy and increased pain reduce activity | Old age has brought about low strength; I’d like to move this table but can’t, I’d prefer to walk hastily but I can’t so I realize that its due to old age, feeling backache as I try to stand up from a latrine which I used to hear my grandmothers talk about but would laugh at them and now, it’s really happening to me. - Female, 66  I have a feeling of how my energy to do work keeps reducing, for example yesterday I worked in my banana plantation, but that alone left me so weak through the whole night. This is a clear sign that my health is really deteriorating. - Male, 62  When I am from digging in my garden after reaching home, I feel so tired that I might even fail to cook for myself lunch - Female, 55 | Generally I no longer do my activities as I used to, because nowadays after digging, I cannot came back home and feel like doing any other thing because I am always tired, I can’t wash, or peel as I used to do, because I used to do all my activities with ease but now I do not, and that means that it all ended with having good health. - Female, 60  My health has changed because I can no longer carry heavy weights. And I would work from morning up to evening, but now days I even sometimes dodge working in the evening. Or I may wake up when my leg or chest is paining me and I don’t work and keep at home the whole day. - Male, 65 |
| Assistance with daily activities is common | I no longer do some of the activities that I used to do when I was still a young adult. For instance, like carrying a big jerry can of water, going to the bush to correct firewood, I cannot walk up to the marketplace, so I wait for my grandchild to come back from school and help me. - Female, 72 | I used to carry a full jerry can of water but now I even struggle carrying a small jerry can of water. And that is why I stay with some children so that they can help me in one way or another. - Female, 73 |
| Fear of noncommunicable diseases | I fear stroke. It’s a very bad condition. There are also people who are diabetic and they end up cutting their legs off. Those are the conditions I pray to God that they should not come to me. - Male, 64  You hear that a person has fainted and died due to pressure yet on the outside he looked all fine. So that’s why I fear it! You will be with a person today in the morning or yesterday and hear that he was hit by pressure and is dead. Sometimes he’s not dead but hit by stroke where he can’t feed himself or raise himself up from the bed. - Female, 53  Cancer…I really fear it. Do you know I keep pressing my body to find out if I will have got it already? Cancer is a bigger enemy than HIV. I also cannot compare cancer with hypertension or diabetes, cancer is way far dangerous. - Female, 60  A pressure and diabetes patient does not accomplish his tasks while an HIV patient will. - Male, 60 | We always viewed HIV/AIDS as a monster. I believe that it’s not feared as much as cancer is today. - Male, 49  I am worried about that condition, because they say that when someone has high blood pressure, he or she can be attacked without any warning signs and can die in a short time. - Female, 57  I don’t want to fall sick of cancer, diabetes and hypertension, because they are not curable. And I usually pray to God to continue protecting me from such diseases. - Female, 49  You see I am worried, because I know that pressure can hit me any time and diabetes can erupt and disrupt my health. - Female, 51 |
| Fear of illnesses that affects physical functioning | Almost all parts of my body are very important features of my health. For instance, I would like to keep with my legs walking normally. There are some people of my age who can no longer walk like me. Their legs are swollen. I therefore pray to God to keep my health in this state. [….] I fear for my body to ever stop functioning the same way, for instance my legs should never get crippled that I may not be able to have my usual travels. - Male, 64  I don’t want to reach an extent where I can’t wash my clothes, cook for myself, failure to close up. - Female, 57 | Living without energy to work, I really don’t want that to happen to me. - Male, 49  When I am seeing something I can be able to do it for myself. Also I want to remain with hands to enable me do my activities plus the head to enable me think before I act. - Male, 56  I pray to God that I remain with my sight because I see darkness sometimes. And without eyes, you cannot perform well your activities because the eye goes direct with hands and feet to perform day to day tasks. - Male, 65 |
| Fear of infectious diseases | Cholera is a disease that kills first, and tuberculosis is also painful, because I have ever suffered from it, so I don’t want to suffer from it again. - Male, 56  I still think about [TB]. Not that I don’t think about any sickness such as headache or malaria, but, TB, I know that it’d kill me. - Female, 55 | When any of your friends tests positive you cannot fail to fear. - Male, 53  I fear getting infected with HIV, because that virus deteriorates someone’s health completely, and so there are some activities you will no longer do when you have HIV, which in turn reduces on the household income, as more money goes to treatment. - Female, 56  HIV [is] a disease that worries me a lot. But nowadays for those who get infected with HIV and they start medication right away, they can no longer have worries about it. - Female, 62 |
| Financial security essential for good ageing | The most poisonous situation is to become needy at an older age. For example, when one cannot even afford to buy him/herself a ‘Headex’ pill. - Male, 64  I’d like to have a plantation, because, even if I cannot help myself, I’d like to have a place that I can have matooke from since you cannot know about the future. But if you weed, acquire bananas from the plantation, sleep in a non-rented house then that’s a good life. - Female, 57 | If you have good health, you can plan, make money and then buy the things you want in life such as a bicycle or motorcycle and look after your family as required. -Male, 59  When he or she has property he has money. This makes one live good life, because he has nothing to worry about. Yes, a problem can happen sometimes, but when it happens one can be able to overcome it when he has money. So when one has property, wealth, livestock, and money then he is living a good life. - Female, 51 |
| Social support of family is preferred | I know by aging one loses energy to up keep himself, and I will need constant help from my family members to look after me, feed me, and keep me clean to be able to live well like them. - Male, 70  When I grow older than now, I would wish to be seated in one place, playing and having fun with my grandchildren as they make me smile, make fun of me, like how I used to play and make fun of my grandparents when I was still young, and I don’t want to keep alone because I feel I will get bored if I keep alone. - Female, 72 | My grandchildren, when I am no longer able to work, I hope they will work for me such that if one brings water I will take, if one brings me porridge I will take. - Female, 65  These children I stay with help me in many ways; I can send them somewhere for something and they help bring it to me. And when my workers are off, I send my grandchildren to look after the goats. - Female, 56  And for my children I want them to always keep checking on me and bring me bread and some money. - Male, 65 |
